# A novel splice donor mutation in *DCLRE1C* caused atypical severe combined immunodeficiency in a patient with colon lymphoma: case report and literature review

**DOI:** 10.3389/fonc.2023.1282678

**Published:** 2023-10-12

**Authors:** Xiaoqing Zhang, Wujun Jiang, Zhongqin Jin, Xueqian Wang, Xiaoxiang Song, Shan Huang, Min Zhang, Huigang Lu

**Affiliations:** ^1^ Department of Medicine, Children’s Hospital of Soochow University, Suzhou, China; ^2^ Department of Prenatal Screening and Diagnosis Center, Affiliated Maternity and Child Health Care Hospital of Nantong University, Nantong, China; ^3^ Department of Clinical Immunology, Children’s Hospital of Soochow University, Suzhou, China; ^4^ Department of Pathology, First Affiliated Hospital of Soochow University, Suzhou, China; ^5^ Department of Pathology, Children’s Hospital of Soochow University, Suzhou, China

**Keywords:** *DCLRE1C*, ARTEMIS, hypomorphic mutation, severe combined immunodeficiency, radiosensitive immunodeficiency

## Abstract

**Introduction:**

Hypomorphic mutations of *DCLRE1C* cause an atypical severe combined immunodeficiency (SCID), and Epstein-Barr virus (EBV)-related colon lymphoma is a rare complication.

**Case presentation:**

A teenage boy presented with colon EBV-related colon lymphoma, plantar warts, and a history of recurrent pneumonia. His peripheral blood lymphocyte count and serum level of immunoglobulin (Ig) G were normal, but he exhibited a T^+^B^-^NK^+^ immunophenotype. Genetic analysis by whole exome sequencing revealed compound heterozygous mutations of *DCLRE1C* (NM_001033855.3), including a novel paternal splicing donor mutation (c.109 + 2T>C) in intron 1, and a maternal c.1147C>T (p.R383X) nonsense mutation in exon 13. Based on his clinical features and genetic results, the diagnosis of atypical SCID with colon lymphoma was established. Our review shows that seven patients, including our patient, have been reported to develop lymphoma, all with hypomorphic *DCLRE1C* mutations. Among these cases, six had EBV-related B-cell lineage lymphoma, and one had Hodgkin lymphoma with EBV reactivation. Unfortunately, all of the patients died.

**Conclusion:**

Recognizing the radiosensitivity of the disease is critical for the prognosis. Hematopoietic stem cell transplantation before being infected with EBV is an optimal treatment.

## Introduction

The nuclease ARTEMIS, encoded by the DNA cross-link repair enzyme 1C (*DCLRE1C*), is involved in double-stranded DNA repair and V(D)J recombination via non-homologous end joining (NHEJ). Null mutations of *DCLRE1C* lead to complete ARTEMIS deficiency and cause T^-^B^-^NK^+^ severe combined immunodeficiency (SCID) with sensitivity to ionizing radiation and usually present in the first year of life (1). Hypomorphic mutations of *DCLRE1C* cause reduced ARTEMIS function and ‘leaky’ SCID, resulting in severe and progressive consequences with an initially indolent and late-onset clinical course. Mutations in *DCLRE1C* occur more frequently in Western countries (2). In China, only several cases with *DCLRE1C* mutations associated with SCID were recently reported (3–6). Here, we described a male Chinese adolescent with a novel hypomorphic mutation who displayed a milder immunodeficiency but progressed to malignant colon lymphoma. This study was approved by the institutional ethics committee of Children’s Hospital of Soochow University (No.2022CS053).

## Case presentation

**Figure 1:**
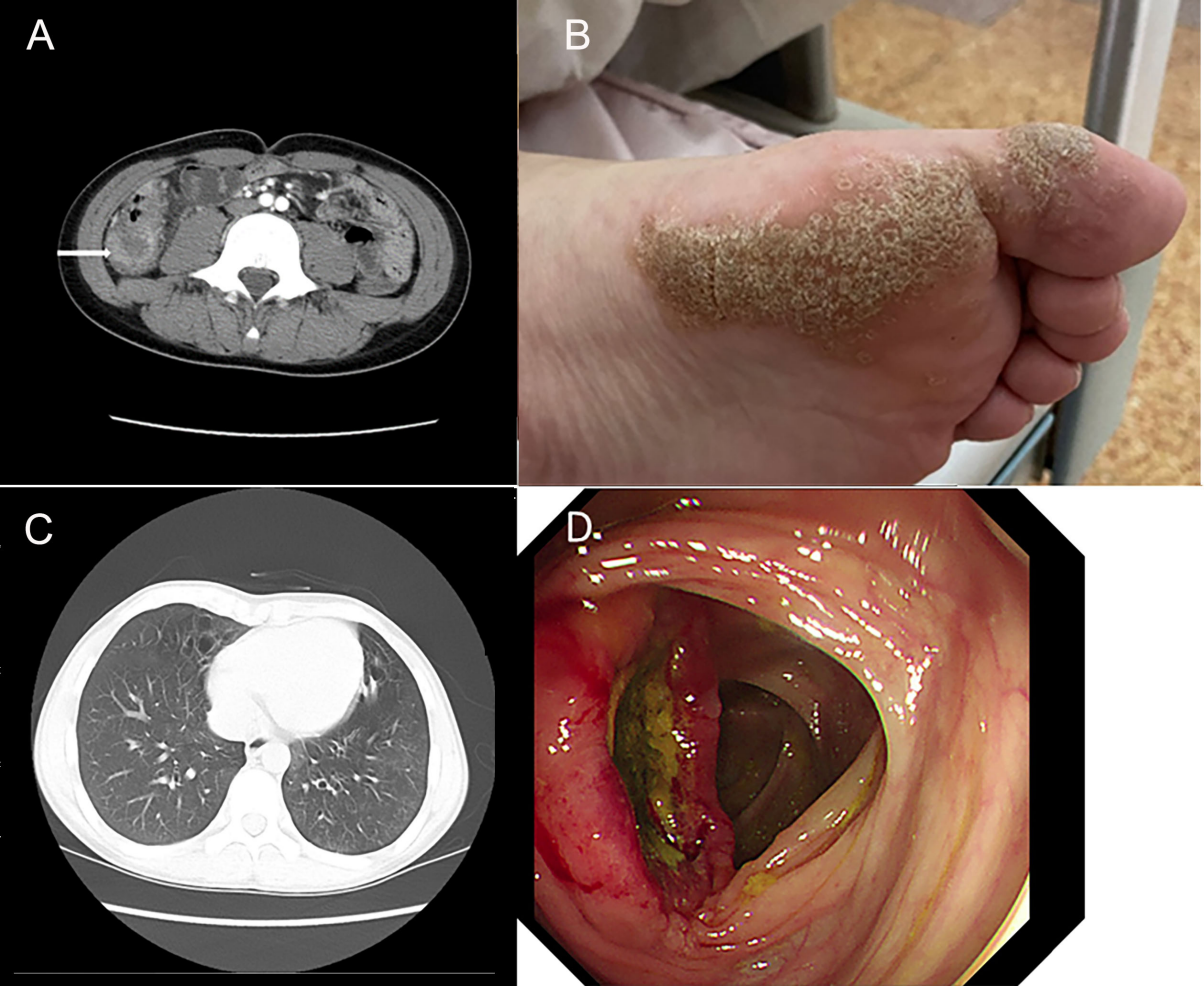
Clinical images show the clinical manifestation of the patient. **(A)** Computerized abdomen tomography (CT) revealed the thickened intestinal wall of ileocecum and patchy blurred shadows around it. **(B)** Skin lesions on the soles of the patient’s left foot. **(C)** Chest CT showed bronchiectasis in both lungs. **(D)** A huge ulcer with yellow pus moss on it of ascending colon on colonoscopy.

**Figure 2:**
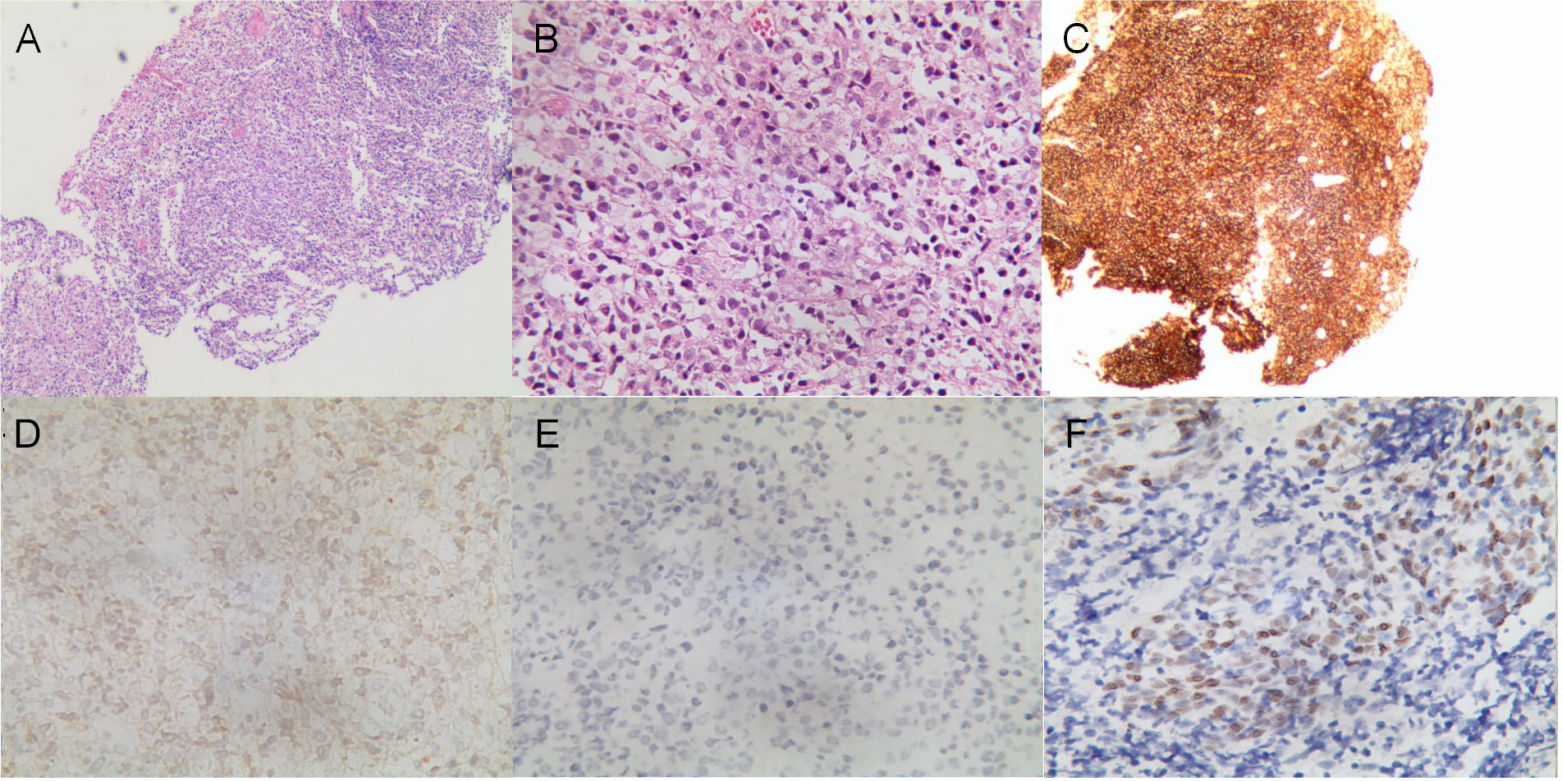
Histological analysis of the mucous membrane of the colonic ulcer. **(A, B)** Diffuse lymphoid proliferation of medium-sized lymphocytes stained with hematoxylin–eosin **(A)** (×40), and **(B)** (×400). **(C–E)** Diffuse CD20 positive lymphoid infiltration **(C)** (×40), sporadically positive for Kappa-light chain **(D)** and negative for Lambda-light chain **(E)** on immunohistochemistry (×400). **(F)** *In situ* hybridization showed EBER positive for lymphoma tumor cells (×400).

A 13.5-year-old male patient with nonconsanguineous parents was admitted to our hospital with a complaint of abdominal pain for one month. The abdominal pain was paroxysmal, located around the periumbilical and right lower quadrants. Three days before being admitted to the hospital, he had loose stools with mucus and blood in a row. His peripheral blood showed a normal level of lymphocytes, neutrophils, erythrocytes, and platelets. Both abdominal ultrasound and computerized tomography (CT) revealed ileocecitis and a small volume of ascites (**Figure 1A**). He had recurrent pneumonia since the first year of life and had no history of transfusions. His sister had oral ulcers and died of severe pneumonia at four months old. Both his father and grandfather had histories of tuberculosis. Physical examination revealed a normal nutritional status, tenderness around the umbilicus and right lower abdomen on palpation, and skin lesions on the soles of the left foot (**Figure 1B**). There were no palpable enlarged lymph nodes or rales of bilateral lungs on auscultation. The number of lymphocytes was 2.63×10^3^/μl. The percentage of CD3^+^ T lymphocytes was 83.48% (reference range: 55-83%), of which CD3^+^CD4^+^ T lymphocytes was 22.69% (reference range: 28-57%), CD3^+^CD8^+^ T lymphocytes was 51.50% (reference range: 10-39%), and CD4^+^/CD8^+^ ratio was 0.44 (reference range: 0.98-1.94). The percentage of B cells was only 0.17%, dramatically lower than the reference range (6-19%), while that of NK cells was normal. Serum levels of immunoglobulin (Ig)M and IgA were low (IgA 0.01 g/L, reference 0.63-3.04 g/L; IgM 0.29 g/L, reference 0.50-2.48 g/L). However, the serum levels of IgG and IgE were normal. The DNA copy number of EBV in blood was determined by quantitative PCR (qPCR) at 3.83×10^3^ copies/ml. Other tests were negative, including cytomegalovirus (CMV) DNA PCR, T-spot, human immunodeficiency virus (HIV) antibody, antinuclear antibodies, antineutrophil cytoplasmic autoantibodies, anti-dsDNA antibody, anti-Sm antibody, anti-SSA/SSB antibody, anti-Ro-52 antibody, anti-ribonucleoprotein (RNP) antibody, anti-Jo-1 antibody, anti-SCL-70 antibody, tumor markers, fecal culture, and fecal Clostridium difficile gene Xpert assay. Chest CT showed bronchiectasis in both lungs (**Figure 1C**), and echocardiography revealed mild aortic valve regurgitation. He received a colonoscopy, during which a massive ulcer with yellow pus moss in the ascending colon was observed (**Figure 1D**). Diffuse proliferation of medium-sized lymphocytes was found in the biopsy from the ulcer (**Figures 2A, B**), the nuclei exhibit clear heterogeneity, appearing basophilic, with the presence of prominent large nucleoli. Immunohistochemical analysis showed the cells were positive for CD20^+^ (**Figure 2C**), CD79a, MUM1, very weakly positive for CD138 and CD68, sporadically positive for CD2, CD3, and Kappa-light chain (**Figure 2D**), and negative for Lambda-light chain (**Figure 2E**). Ki-67 revealed a proliferative fraction of 60% of the cells. EBV-encoded mRNA (EBER) *in situ* hybridization was tested positive (**Figure 2F**), and acid-fast staining was negative. Transplacental maternal lymphocytes were excluded by short tandem repeat analysis. Combined with his past history of recurrent respiratory tract infections, nearly deficient B cells, and positive family history, a clinical diagnosis of primary immunodeficiency, colon lymphoma, and plantar warts was determined.

**Figure 3:**
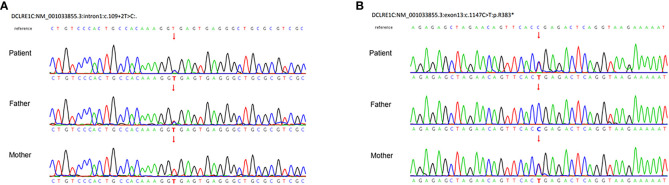
Sanger sequences demonstrated compound heterozygous mutations of the *DCLRE1C* gene. **(A)** A paternal splicing donor mutation (c.109 + 2T>C) in intron1. **(B)** A maternal c.1147C>T (p.R383X) nonsense mutation in exon13 (NM_001033855.3).

After a consent form was signed, trio-whole exome sequences (WES) were performed. We prioritized variants that were previously reported, considered loss-of-function (nonsense, frameshift, or splice sites mutations) or absent in gnomAD. The genetic analysis demonstrated compound heterozygous mutations of *DCLRE1C*, including a paternally inherited splicing donor mutation (c.109 + 2T>C) in intron 1 and a maternally inherited c.1147C>T (p.R383X) nonsense mutation in exon 13 (NM_001033855.3). Sanger sequencing confirmed the carrying status of variants (**Figure 3**). There are only two carriers in gnomAD of c.1147C>T (PM2_Surpporting). Since c.1147C>T is located at the 3’-most 50bp of the penultimate exon, nonsense-mediated RNA decay may not occur (7, 8). But the truncated protein loses part of the β-CASP domain (156-385) and the entire C-terminal domain (386-692), which account for 44.7% of the whole protein (PVS1-Strong) (7). Four typical SCID patients with homozygosity for c.1147C>T were identified (9–11) (PM3). Functional study *in vitro* revealed that the recombination and DNA repair activity of p.R383X were decreased (12) (PS3_Surpporting). The patients of the previous and our study carrying c.1147C>T exhibit immunodeficiency which matched the disease phenotype (PP4). In all, c.1147 C>T was classified as likely pathogenic by PM2_Supporting, PVS1-Strong, PM3, PS3_Surpporting, and PP4. c.109 + 2T>C is absent in gnomAD (PM2_ Supporting) and the canonical splicing donor of intron 1 (PVS1_Moderate). It is in trans with c.1147C>T in our case with confirmed paternity and maternity (PM3). The immunodeficient nature of our patient matched the disease characteristics (PP4). In all, c.109 + 2T>C is classified as likely pathogenic by PM2_Supporting, PVS1- Moderate, PM3, and PP4.

The patient was transferred to a different hospital, where further examination confirmed the colon lymphoma to be of the B-cell non-Hodgkin lymphoma variety. He underwent chemotherapy and, tragically, succumbed on the very day he received hematopoietic stem cell transplantation, when it was 1.5 years since the diagnosis of colon lymphoma.

## Discussion

*DCLRE1C*, located on chromosome 10p13, consists of 14 exons. ARTEMIS comprises 692 amino acids, including the highly conserved β-lactamase and β-CASP domains in the N-terminal part (amino acid 1-385) that serves as the enzymatically active motif, and the C-terminal domain that modulates the nuclease activities. Nevertheless, nonsense-mediated RNA decay is not predicted to occur for the c.1147C>T mutation. The truncated protein p.R383X loses part of the β-CASP domain (156-385) and whole C-terminal part domain (386-692). According to recent studies, this nonsense variant encodes function-null variants, as in typical SCID patients with homozygous c.1147C>T mutations, whose *DCLRE1C* mRNA is intact but lacks V(D)J recombination and double-strand breaks (DSB) DNA repair (9). The novel paternal variant c.109 + 2T>C is a canonical splice-site mutation in intron 1. The splice-site mutation at the adjacent nucleotide position (c.109 + 1G>T) was reported to generate some canonical wild-type transcripts, implying that it is associated with a late-onset and less severe phenotype (13). Therefore, it is reasonable to deduce that the novel splice site mutation c.109 + 2T>C is a hypomorphic variant, leading to the leaky phenotype in the patient.

In our case, the counts of absolute lymphocyte number (ALN) and CD3^+^ lymphocytes were within the reference range, and maternal engraftment was ruled out. He had a B-cell deficiency with surprisingly normal IgG and IgE serum levels. Similar laboratory findings were found in other patients with hypomorphic *DCLRE1C* mutations (14), whose naïve T cells decreased, indicating that T cell development was affected by the hypomorphic mutations; compensatory proliferation might have caused the normal peripheral T cells due to residual V(D)J recombination. The central compartment of B cell progenitors was thought to be more prone to depletion than the T cell compartment (14), despite the fact that ARTEMIS was proposed to play equal roles in recombination for T and B cells. Since ARTEMIS participates in NHEJ, which is involved in the Ig class switch, normal serum levels of his IgG and IgE support the presence of a residual functional B cell compartment and V(D)J recombination.

We searched on PubMed using the keywords ‘severe combined immunodeficiency,’ ‘lymphoma,’ and ‘*DCLRE1C*’ or ‘ARTEMIS’ for articles published from 2001 to 2023. We reviewed the genetic and clinical features of patients with ARTEMIS deficiency complicated by lymphoma (**Table 1**). Seven patients, including our patient, have been reported to develop lymphoma, all with hypomorphic *DCLRE1C* mutations (15–18). Six patients had EBV-related B-cell lineage lymphoma, and one had Hodgkin lymphoma with EBV reactivation. The average age of lymphoma onset was 11.75 years old (ranging from 9 months to 23 years old), with a male-to-female ratio of 4:3. Our patient is approximately at the average age of tumorigenesis. The mechanisms of lymphoma in these patients remain unclear. Since most of them were EBV-related B-cell lineage lymphomas, no lymphoma has been found in null mutation patients. One explanation for this is that EBV mainly infects B cells, and patients with non-functional *DCLRE1C* mutations cannot establish infection. Additionally, most patients may have died before getting infected with EBV (19). Besides, patients with reduced functional mutations lack sufficient CD8^+^ cytotoxicity against EBV infection, resulting in EBV-driven B cell colony proliferation (19). Lastly, the partial activity of ARTEMIS results in impaired NHEJ, which plays a vital role in DNA DSBs and is associated with profound genomic instability. This has been documented in cells from two of these patients (P1 has a clonal trisomy of chromosome 9, and P2 has a translocation of Chromosome 7 and 14). And genomic instability is known to be susceptible to malignancy (20).

**Table 1:** Genetic and clinical characteristics of patients with ARTEMIS deficiency complicated with lymphoma.

Patient	gender	origin		Age of diagnosis of lymphoma	mutations	Lymphocyte counts and subpopulations	PHA responses	Immunoglobulins	Cytogenetic analysis	Sites of involvement	EBV-associated	Treatment	Outcome	Ref.
P1	*M*	*France*		*9m*	Compound heterozygous del. Exon1-3*, *del. T1384–A1390*	*ALN:66/ul↓↓* *CD^3+^:8/ul↓↓* *CD^19+^:8/ul↓↓* *CD^16+^CD^56+^:18/ul↓↓*	35.8±22cpm× 10^–3(^normally >40^)^	*IgG:14.4* g/L*↑(9months)* *IgA:0.18* g/L*↓* *IgM:6.11* g/L*↑*	*Trisomy, Chr. 9*	*Cervical lymph node, liver, lung, striated muscle*	*Yes*	anti–B cell-specific mAb	Died (5 days after diagnosis)	(15)
P2(sister of P1)	*F*	*Fran*c*e*		5*y*	*Same as* P*1*	*ALN:500-1100/ul↓* *CD^3+^: 280-580/ul↓* *CD^19+^: 8-110/ul↓* *CD^16+^CD^56+^::227/ul*	*26.6 ± 18* cpm× 10^–3(^normally >40^)^	*IgG:1.33* g/L*↓(10months)* *IgA:<0.07* g/L*↓* *IgM:0.56* g/L	*Translocation, Chr. 7:14*	*liv*e*r*	*Y*e*s*	HSCT and anti–B cell-specific mAb	Died(diagnosed 38days after HSCT,12days later died despite treatment with anti–B cell-specific mAb)	(15)
P3	*M*	*Turkey*		*23y*	*H*o*mozygous c.632G>T*	*ALN:800/ul↓* *CD^4+^:177/ul↓* *CD^8+^:277/ul* *CD^19+^: <5/ul↓↓*	*Nr*	*IgG:6.1* g/L*↓* *IgA:0.4* g/L *IgM:0.1* g/L*↓*	N*r.*	*Neck, masseter muscle, lung, spleen, gastroventricular muco*s*a*, a*drenal gland*,	*Yes*	*Chemotherap*y*, radiation*, *HSCT*	Died (of multiple infections)	(16)
P4	*F*	*Israel*		*13y*	*Homoz*y*gous c.1299_1306dup*	*CD^3+^:2200/ul* *CD^4+^:600/ul* *CD^8+^:1300/ul↑* *CD^19+^:350/ul* *CD^16+^CD^56+^:1600/ul↑*	*<20%↓*(stimulation index % of control)	*IgG:1950mg/ml↑* *IgA: <17 mg/ml↓* *IgM:504 mg/ml↑*	N*r.*	*Retrope*r*ito*n*eal lymph node*s	Ye*s*	r*i*t*uximab*	Died (several hours after using rituximab)	(17)
P5	*M*	*Israel*		*15y*	*Same as P5*	*CD^3+^:410/ul↓* *CD^4+^:210/ul* *CD^8+^:180/ul* *CD^19+^:<10/ul↓↓* CD*^16+^* CD*^56+^*:30*/ul↓*	*73%*(stimulation index % of control)	*IgG:1660 mg/ml↑* *IgA: <22 mg/ml↓* *IgM:192 mg/ml↑*	N*r.*	M*edi*a*stin*um, *li*v*e*r	*Yes*	N*r.*	Died (of respiratory failure before chemotherapy or HSCT)	(17)
P6	*F*	*Turkey*		*12y*	*Homoz*y*gous c.194C*>T	*ALN: Normal* *CD^19+^:0.4%↓↓* *CD^20+^:0.7%↓↓* *CD^4+^: 14.7%↓*	N*r.*	*IgG:1070mg/dl* *IgA: <8.2mg/dl↓* *IgM:163 mg/dl*	N*r.*	Lymph nodes in the right cervical and infraclavicular regions	Hodgkin lymphoma with EBV reactivation	*Chemotherap*y*,radiotherapy*	Died (of delayed radiation myelopathy)	(18)
P7	M	China		13.5y	Compound heterozygousc.109+2T>C, c.1147C>T*	ALN:2630/ul CD3+:2195/ul CD4+:597/ul CD8+:1354/ul↑ CD19+:4/ul↓↓ CD16+CD56+:420/ul	Not done	IgG:7.75 g/L IgA:0.01 g/L↓ IgM:0.29 g/L↓ IgE:2.50IU/ml	Not done	colon	Yes	Chemotherapy, *HSCT*	Died (on the very first day of HSCT)	

*null mutations; PHA, Proliferations of phytohemagglutinin; ALN, absolute lymphocyte number; mAb, monoclonal antibody; HSCT, hematopoietic stem cell transplantation; Nr, Not report.

The outcome of these lymphomas is poor. None of these patients were alive despite multiple managements. Three patients succumbed before hematopoietic stem cell transplantation (HSCT). Three patients underwent HSCT, but died afterward. One patient died of delayed radiation myelopathy as an adverse effect of radiotherapy. Three patients died after anti–B-cell-specific monoclonal antibody treatment. Patient P5 surrendered to respiratory failure before further treatment, while patient P3 succumbed to multiple infections (Pneumocystis jirovecii pneumonia, Herpes simplex virus, Staphylococcus aureus, and Candida albicans) 5 months after HSCT (16). Pavel et al. reported a 14-year-old boy with a radiosensitive defect, normal lymphocyte subpopulations, abnormal lymphocyte phytohemagglutinin A, hypogammaglobulinemia, and a heterozygous loss of exon 11 in *DCLRE1C* was diagnosed with B-cell non-Hodgkin lymphoma. He survived after receiving HSCT with reduced-intensity conditioning regimens, and no EBV infection was mentioned (21). Thus, EBV infection could be fatal in this group of patients. The high mortality of this disease calls for an early diagnosis before patients being EBV infected. Patients with radiosensitive CID have a higher priority to receive a curative therapy, HSCT. However, the survival rate has been reported to be lower than other types of CID (22). Recognizing the radiosensitive nature of the disease is quite essential. Radiomimetic drugs, such as alkylating-containing regiments, and radiation therapy should be tailored in pre-and post-transplant treatment to reduce related toxicity and optimize the prognosis (21). The most recent findings on lentiviral gene therapy offer a promising avenue for treating this illness (23). Nevertheless, the impact on a greater quantity of cases and the safety of its application to patients with EBV infection remain uncertain.

In conclusion, we reported a leaky SCID with heterozygous *DCLRE1C* mutations, including a novel splice mutation. This is the first reported case of atypical SCID associated with a hypomorphic *DCLRE1C* mutation in Chinese, presenting an uncommon complication of colon lymphoma. Leaky SCID should be considered in patients with a late-onset of the disease and a lack of immune cells or immunoglobulins. The combination of leaky SCID and a history of malignancy indicates potentially radiosensitive immunodeficiency. Genetic detection and radiosensitivity testing are crucial for diagnosis. HSCT before being infected with EBV should be considered as an optimal option in clinics.

## Data availability statement

The original contributions presented in the study are included in the article/supplementary material. Further inquiries can be directed to the corresponding author.

## Ethics statement

Written informed consent was obtained from the individual(s), and minor(s)’ legal guardian/next of kin, for the publication of any potentially identifiable images or data included in this article.

## Author contributions

XZ: Conceptualization, Data curation, Investigation, Methodology, Resources, Writing – original draft, Writing – review & editing, Validation, Visualization. ZJ: Conceptualization, Data curation, Investigation, Methodology, Visualization, Writing – original draft. XW: Data curation, Investigation, Methodology, Writing – original draft. XS: Data curation, Investigation, Methodology, Project administration, Resources, Writing – review & editing. SH: Data curation, Investigation, Resources, Writing – review & editing, Supervision. WJ: Data curation, Investigation, Writing – review & editing, Software, Visualization. MZ: Investigation, Writing – review & editing, Conceptualization, Methodology, Resources. HL: Conceptualization, Investigation, Methodology, Resources, Writing – review & editing, Data curation, Project administration, Supervision, Writing – original draft.
